# Supplementary material for: Predicting corporate credit risk: Network contagion via trade credit
Source: PLoS One. 2021 Apr 29;16(4):e0250115. doi: 10.1371/journal.pone.0250115 (PMC8084139; doi:10.1371/journal.pone.0250115)
Supplement: S1 Appendix — (PDF) [file pone.0250115.s001.pdf]

Supporting Information for Predicting Corporate Credit Risk: Network Contagion via Trade Credit

Claudia Berloco*[1,2], Gianmarco De Francisci Morales*[3], Daniele Frassineti‡[4], Greta Greco[1], Hashani Kumarasinghe‡[5], Marco Lamieri*[1], Emanuele Massaro[3], Arianna Miola[6], Shuyi Yang[1,2]

1 Intesa Sanpaolo, Torino - Italy
2 Università degli Studi di Torino, Torino - Italy
3 ISI Foundation, Torino - Italy
4 Novartis Farmaceutica, Barcelona - Spain
5 Zone24x7, Colombo - Sri Lanka
6 Intesa Sanpaolo Innovation Center, Torino - Italy

* claudia.berloco@intesasanpaolo.com, gdfm@isi.it, marco.lamieri@intesasanpaolo.com
‡ Work done while at Intesa Sanpaolo.

Temporal aggregation

Jaccard similarity

As previously stated, we generate the networks at time t by aggregating information of the transactions between two firms for the previous $t^* = t - 12$ months. However, during the exploration phase of the dataset we tested different aggregation windows, i.e., $t_r = 3, 6, 12$ months, and we generated different transactions networks according to them. Our findings reveal that the difference by using different temporal aggregation is negligible in both the structure of the networks and the dynamics of the label propagation. In order to understand the similarity between the aggregated networks, we report the Jaccard similarity index between between the networks (Fig S1). The Jaccard index is a statistic used for comparing the similarity of two sets, and is defined as the size of the intersection divided by the size of the union of the sample sets:

$$J(A, B) = \frac{|A \cap B|}{|A \cup B|} \tag{1}$$

We report the results of the distribution of the average value of the Jaccard index for the edges of networks aggregated every 3-6-12 months. In the first scenario (Fig S1 left) we performed a 1-month sliding window aggregation. For instance we aggregated the transactions for the months of Jul-Aug-Sep and compared with the network of aggregated transactions of the months of Aug-Sep-Oct, and so on. While in the second scenario (Fig S1 right) we use a tumbling window: in this case we compare the months of Jul-Aug-Sep with the months of Oct-Nov-Dec. The results suggest that different temporal aggregations do not significantly affect the similarity of the networks.

Spatial autocorrelation

We also analyzed the spatial autocorrelation of some variables using different temporal aggregation. Spatial autocorrelation pertains to the non-random pattern of attribute values over a set of spatial units (the network in our case). This pattern can take two general forms: positive autocorrelation, which reflects value similarity in space, and

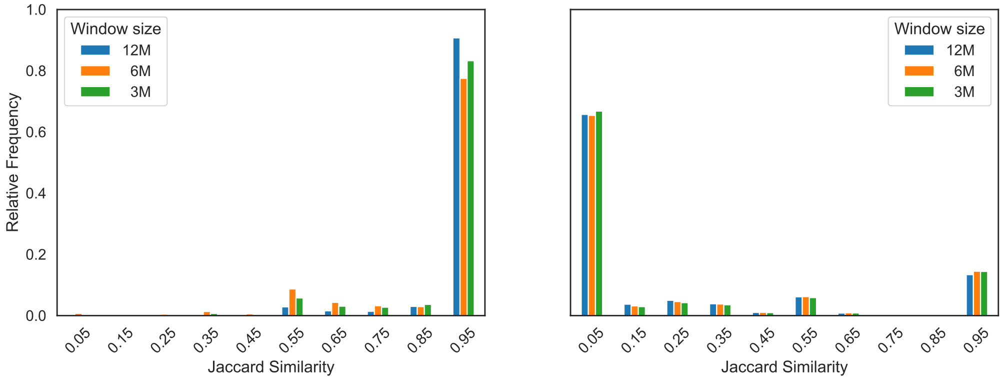

Fig S1. Distribution of the Jaccard index (1-month sliding window aggregation on the left, tumbling window on the right) for different temporal aggregation window: 12 months (blue), 6 months (green), and 3 months (orange).

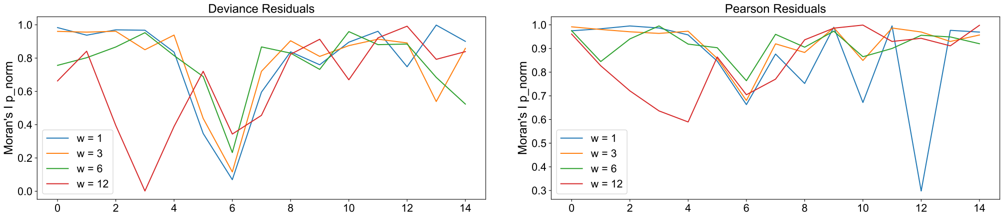

Fig S2. p-values of the Deviance and the Pearson residuals for Moran's I spatial autocorrelation, for networks with weights corresponding to fraction of revenues between adjacent nodes.

negative autocorrelation, or value dissimilarity in space. In either case the autocorrelation arises when the observed spatial pattern is different from what would be expected under a random process operating in space. Spatial autocorrelation can be analyzed from two different perspectives. Global autocorrelation analysis involves the study of the entire map pattern and generally asks the question as to whether the pattern displays clustering. Local autocorrelation, on the other hand, shifts the focus to explore within the global pattern and identify clusters or so called 'hot spots' that may be either driving the overall clustering pattern, or that reflect heterogeneities that depart from global patterns.

In what follows, we highlight the global spatial autocorrelation by considering Moran's and Geary's measures, implemented in the PySAL library.[1]

Moran's I Moran's I measures the global spatial autocorrelation in an attribute y measured over n spatial units and is given as:

$$I = \frac{n}{S} \frac{\sum_i \sum_j z_i w_{i,j} z_j}{\sum_i z_i z_j}, \tag{2}$$

where $w_{i,j}$ is a spatial weight, $z_i = y_i - \bar{y}$, and $S_0 = \sum_i \sum_j w_{i,j}$.

Geary's C Geary's C is a similar measure defined as follows:

$$C = \frac{(n-1)}{2S_0} \frac{\sum_i \sum_j w_{i,j}(y_i - y_j)^2}{\sum_i z_i^2}. \tag{3}$$

[1] `https://pysal.readthedocs.io/en/latest/users/tutorials/autocorrelation.html`

We consider aggregated networks by different time windows (3, 6, 12) and different metrics to analyze the spatial autocorrelation: Deviance Residuals (Rd) and Pearson Residuals (Rp). In Fig S2 we show an example of the results of the p-value of the Moran's I for the case of the Deviance and Pearson residuals for networks with weights corresponding to fraction of revenues between the adjacent nodes. The most relevant result is that the temporal aggregation does not have a relevant effect on the spatial correlation.

Cascade analysis

For this analysis, we draw inspiration from the seminal work on information cascades by Kempe et al. [1] to model the default contagion process in our inter-firm network. In particular, we assume the contagion follows an *independent cascade* process on the transaction network. When a node u becomes active (i.e., experiences a default), it has a single chance of activating each inactive neighbor v, with some probability p_{uv}. Given a set of activations, the inference problem requires reconstructing the most likely diffusion path for the cascade. However, here we are interested in analyzing all the *possible* diffusion paths, which represent an upper bound on the information content of the network.

For example, assume a simple network composed of three nodes u, v, w as the one in Fig S3. Both u and v activate at time $t_1 < t_2$, while w activates at time t_2. Node w has incoming edges from both u and v. According to the independent cascade model, both u and v may be responsible for the activation of w. Thus, we define a *potential cascade* (henceforth simply referred to as a cascade) as the union all the possible diffusion paths in the graph. Each edge in the cascade is a possible diffusion vector for the default contagion process.

Fig S3. An example of a default cascade.

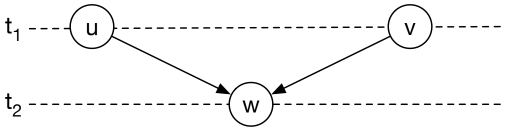

Note that each cascade is a directed acyclic graph (DAG), as the arrow of time allows us to break possible cycles. We also assume that if two firms activate at the same timestamp, then they do not influence each other. That is, the diffusion can only happen from an activation strictly in the past. Given that the temporal dynamic of the process is not know in advance, we do not impose any limit on the time delay between two activations (i.e., once a node is active, it is contagious for the rest of the timeline). We find 595 distinct (disconnected) cascades by this process, for a total of 4340 nodes and 4499 edges.

Figures S4 and S5 show the distribution of the number of nodes and edges for the set of cascades inferred from the dataset. The distributions are extremely skewed, with the largest cascade dwarfing all the others, therefore we also show the same graphs with the largest cascade excluded. Most of the cascades are rather small, with the bulk consisting of only two nodes and a single edge.

We define the depth of a cascade as the maximum shortest-path distance between the root node of the DAG and the rest of the nodes. The depth of a cascades gives us an indication of how far (i.e, how many hops in the graph) the diffusion process can reach. The distribution of the depth of the inferred cascades is shown in Fig S6a and summarized in Fig S6b. Apart from the largest cascade, which has depth 9, all the other cascades have a maximum depth of 3, with most having a depth of 1. Therefore, the diffusion process as modeled seems to be fairly localized.

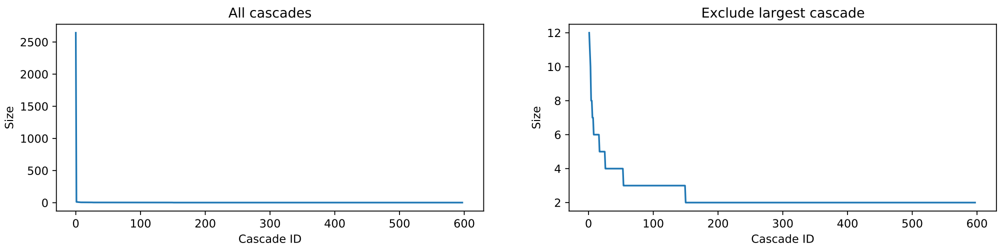

Fig S4. Distribution of the number of nodes (infected) per cascade.

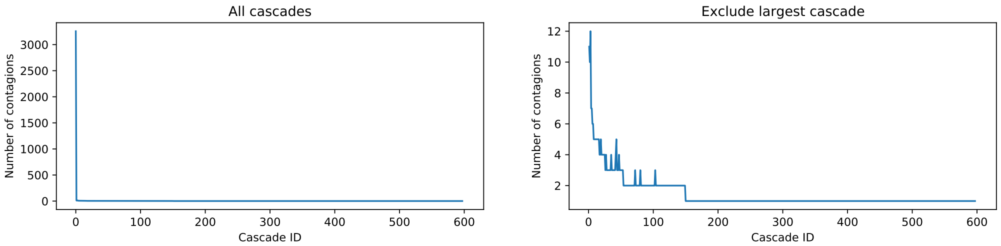

Fig S5. Distribution of the number of edges (possible contagions) of the cascades.

We now look at the time difference between two activations in the cascades (δ_t) to get an idea of the possible temporal dynamics of the diffusion process. Fig S7 shows the distribution of the difference between the activation times for each pair of nodes connected by an edge in the cascades. We can see a mostly linear decay for the ≈2 years observation window: it would seem most contagions are more likely to happen in a short delta of time. However, upon closer inspection, the linear decay in probability is actually compatible with a window effect. To observe an edge in the cascade both activations should fall within the observation window. The larger the time delta between these two observations, the lower the likelihood that both fall within the window ($p \propto \frac{W-\delta_t}{W}$, where W is the size of the observation window).

Thus, we do not see any decaying effect of time on the contagion probability in the dataset under study. The horizon of the temporal dynamic of the process is possibly much larger than 2 years. As we show in the next section, experiments with a prediction feature based on this analysis confirm the absence of linear temporal decay in the contagion process.

Finally, we wish to understand what are the upper bounds of an application of a cascade-like model in a prediction setting. To do so, we build a simple *prediction oracle*. The oracle works as follows: for each firm in the dataset, if at timestamp t the firm has target variable $Y = 1$, if the firm is present in a cascade and has in-degree larger than zero, then the oracle predicts output $\widehat{Y} = 1$, otherwise $\widehat{Y} = 0$. This condition captures the fact that the network-based oracle can foresee with certainty the contagions before

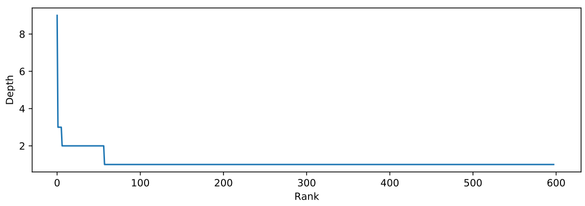

(a) Distribution of the depth of the cascades.

Depth	Number of cascades
1	544
2	47
3	3
9	1

(b) Number of cascades with a given depth.

Fig S6. Depth of the observed cascades.

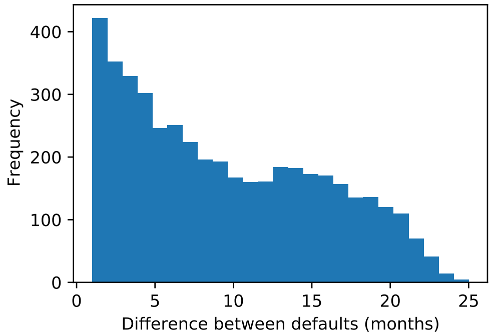

Fig S7. Distribution of the time difference between activations of two nodes connected by an edge in any of the cascades.

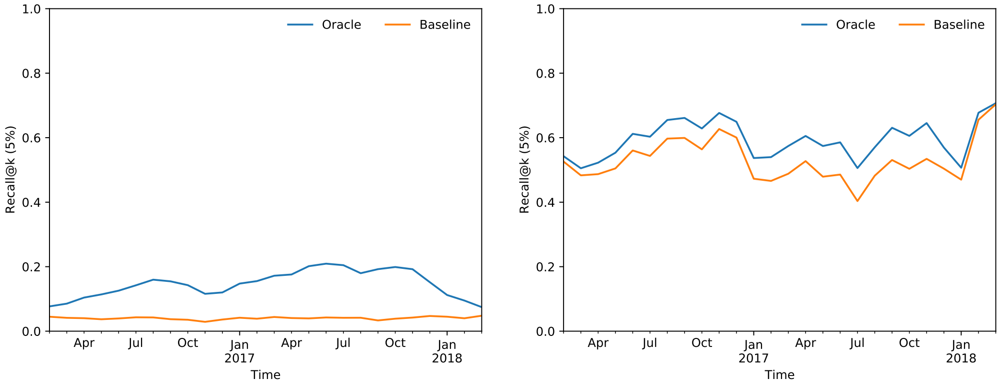

(a) Oracle based on the default cascades compared to a random baseline.

(b) Logistic regression model (baseline) compared with one enhanced by oracle predictions.

Fig S8. Recall@K for different cascade-based oracles.

they happen, but cannot foresee the first activation.

Fig S8a shows the performance of the oracle compared with a simple random guess, which just sorts the firms randomly. The average recall at K (R@K, $K = 5\%$) for the random baseline is around 4% while the oracle gets an average of 14%. This marked improvement represents an upper bound on how much we can expect from a purely contagion-based model. Fig S8b shows the results for a similar setting, where the baseline is a logistic regression (LR) model trained only on features of the firm, and the oracle enhances the predictions for the firms present in a cascade with in-degree larger than zero. The average R@K for the baseline LR model is 53%, while the oracle averages 59%. This result confirms that the network information has the potential to improve the performance for the default prediction task.

Financial statement items

Tables S1 and S2 report a list of the financial statement items available in the dataset during the development of our models.

Table S1. Income statement

Net Sales
Cost Of Goods Sold
Personnel Expenses
Other Operating Expenses
Gross operating income (EBITDA)
Depreciation and Amortization
Operating Income (EBIT)
Interest and Related Expense
Financial Losses
Interest Income
Dividend Income
Net Interest Expenditures
Net Other Non-operating Income/Expenses
Income / (Loss) from Affiliates
Impairment of Financial Assets
Extraordinary Revenues and Expenses
Earning before Taxes (EBT)
Income Taxes
Minority Interest
Total Net Income (after minority interest)

Table S2. Balance sheet

**ASSETS**	**LIABILITIES**
Property, plant & Equipment	Long Term Borrowings (>12 months): [D]
Capitilised Development costs	Retirement and other emploee long-term benefit provisions
Goodwill	Risk and charges provisions(>12 months)
Other intangible Assets	Deferred taxation provisions
Investment in associates	Other Long-term liabilites(¿12 months)
AFS - investments	Non - Current Liabilities (SUBTOTAL)
Deferred Tax Assets	Trade Payables (>12months)
Long term Loan Receivable	Trade Payables (< 12months)
Other Non Current Assets	Employee and Social Security payables
Non - Current assets (SUBTOTAL)	Short - Term financial Borrowings (< 12 months): [E]
Inventories	Current Portion of Long Term Borrowings [L]
Trade Receivables	Risk and charges provisions(< 12 months)
Trading Asset: [A]	Other Long-term liabilites(< 12 months)
Cash funds (cash & cash equivalent): [B]	Current Liabilities (SUBTOTAL)
Other Current Assets	Share Capital
Current assets (SUBTOTAL)	Share Issue premium
Liquidity: [C] = [A]+[B]	Fair Value and other reserves
Total Assets (TOTAL)	Retained earnings and net income of the year
	Equity attributable to Equity holders of the parent (SUBTOTAL): [F]
	Share capital and reserves
	Retained earnings
	Minority interests (SUBTOTAL): [G]
	Total Shareholders' Equity: [H]=[F]+[G]
	Total Liabilities and Shareholder Equity (TOTAL)
	Net Financial Position [I]=[D]+[E]+[L]-[C]

References

1. Kempe D, Kleinberg J, Tardos É. Maximizing the spread of influence through a social network. In: Proceedings of the 9th SIGKDD international conference on Knowledge Discovery and Data mining. ACM; 2003. p. 137–146.
