# Supplementary material for: Predicting corporate credit risk: Network contagion via trade credit
Source: PLoS One. 2021 Apr 29;16(4):e0250115. doi: 10.1371/journal.pone.0250115 (PMC8084139; doi:10.1371/journal.pone.0250115)
Supplement: Attachment — Submitted filename: rebuttal.docx [file pone.0250115.s002.docx]

Response to Reviewers

We thank the editor for handling our manuscript, we welcome the comments of the reviewers, and are grateful for the opportunity to revise and improve our manuscript.

Here a point-by-point list of answers to the questions raised in the reviews, and changes made to the paper to address them.

To the Editor:

1. We have added a funding statement that identifies the industrial affiliations of some of the authors and the role of the funder to the cover letter.
2. We have added our competing interests statement, which already listed the industrial affiliations of some of the authors and declares that “This does not alter our adherence to  PLOS ONE policies on sharing data and materials”, to the cover letter.
3. We have removed the figures from the main file.
4. The supporting information, which is an appendix section, already contains captions for all the figures contained in it. We have separated the appendix in the supporting information, and added a section reference to it at the bottom of the main file.

To Reviewer 1:

1. Fixed typos and poorly worded sentences.
2. Clarified the first sentence of the abstract.
3. Re-plotted Figures 2, 3, 6, 7, 11, S1, S7, S8.
4. Clarified that we present the hybrid model, and the network model is propaedeutic to the final one. Changed the intro to reflect this fact.
5. Clarified that we do not remove additional features in the human validation, it was poorly worded, we simply substitute features for better interpretation. Fixed the text.
6. We checked the performance drop of the RF model in 2017Q4 (November), but we did not find any systematic feature of the dataset that is clearly responsible for the drop (in terms of feature/class distribution). The hybrid model, however, does not seem affected by it, and the performance is in line with the other periods.

To Reviewer 2:

1. Added a section explaining the data we use from the various sources and which risk measures the bank already computes, according to the Basel framework.
2. Added a section on Model-based approaches that explains the most used models for bankruptcy modeling and credit risk, which explains what an Early Warning System is, and added the relevant citations.
3. Added a benchmark of a standard model for credit risk (Altman’s model) and compared to it in Figure 11.
4. Re-plotted figures in vectorial format to avoid blurriness.
5. Improved the flow of the paper, we hope the message is clearer now.

We have also polished the text and improved the flow of the paper. We hope that the revised and improved paper addresses the main concerns of the reviewers.

Best Regards,

Claudia Berloco,Gianmarco De Francisci Morales, Daniele Frassineti, Greta Greco,
Hashani Kumarasinghe, Marco Lamieri, Emanuele Massaro, Arianna Miola, Shuyi Yang
